# Immunological and histopathological changes in sheep affected with cutaneous squamous cell carcinoma and treated immunotherapeutically

**DOI:** 10.14202/vetworld.2017.1094-1103

**Published:** 2017-09-20

**Authors:** Faten A. M. Abo-Aziza, A. A. Zaki, A. El-Shemy, Sahar S. Abd Elhalem, Amany S. Amer

**Affiliations:** 1 Department of Parasitology and Animal Diseases, Veterinary Research Division, National Research Centre, Cairo, Egypt; 2 Department of Physiology, Faculty of Veterinary Medicine, Cairo University, Giza, Egypt; 3 Department of Zoology, Women Faculty for Arts, Science and Education, Ain Shams University, Cairo, Egypt

**Keywords:** antibody, cutaneous squamous cell carcinoma, histopathology, immunotherapy, sheep

## Abstract

**Background and Aim:**

Recently, it has been recorded unexpected percentage of cutaneous squamous cell carcinoma (cSCC) in sheep. Despite the improvement in surgical treatment, the outcome of animals remains limited by metastatic relapse. Although antibodies for cancer treatment have been practiced for many decades, the use of this methodology in animals is deficient. This study aimed to establish cSCC therapy by tumor cell protein antibody (Ab1) or secondary antibody (Ab2) raised by two series of immunization in the same strain of rabbits.

**Materials and Methods:**

A total of 19 Ossimi sheep were used (14 sheep suffered from cSCC and 5 were apparently healthy). Each animal from control healthy group (n=5) and control cSCC (n=4) group was treated with a course of eight injections of normal globulins. Animals in the third (n=5) and the last (n=5) groups received a course of eight injections of Ab1and Ab2, respectively. Each tumor was measured before and after treatment. The eight injections were applied at 1^st^, 3^rd^, 5^th^, 7^th^, and 9^th^ week and the remaining three injections were at 1 week interval. Tissue specimens and blood samples were taken for histological and immunological studies.

**Results:**

The obtained results revealed that injection of Ab1 might prevent the bad prognostic picture of polymorph infiltration without any criteria of regression % of tumor. Treatment with Ab2 showed regression of tumor size ranged between minimum of 8.99% and maximum of 78.12%, however, the measurements in most cases reached the maximum regression after the past two injections. In additions, infiltration of lymphocytes to tumor site, normalization of leukocytes picture and also increase of antibody titer were observed.

**Conclusion:**

This profile might confirm that Ab2 could act as an antigen and encourage us to use it as a tumor vaccine. Extensive studies are needed to isolate the idiotypic portion of Ab1 for raising Ab2 as an anti-idiotypic antibody to be used as tumor vaccine. The question of how lymphocyte traffic to the tumor site as a result of Ab2 injection needs further investigation.

## Introduction

Skin tumors are not only dangerous for humans but also a significant problem in the animal populations. Cattle, horse, camel, sheep, and goats all suffer from cancer [1-4]. Over the past decades, a worldwide increased in skin tumor to near epidemic proportions has led to high morbidity and economic loss [5]. Risk factors have been well known including ultraviolet radiation, genetic aberrations, chemical carcinogens, and immunosuppression [6,7]. Cutaneous squamous cell carcinoma (cSCC) has been a common, local lesion and sometimes could be invasive to the circulation in most domestic species [1]. cSCC has been reported to be seen in head, back, groins, udder, and perineal regions of adult sheep and goats [8]. Excision was the primary interference option for most cSCC but the complete recovery from tumor was depending on many factors such as location and size of the tumor [9]. Despite the improvement in detection and surgical therapy in the recent years, the outcome of animals remains limited by metastasis relapse [10]. Moreover, the surgical intervention was not the ultimate solution to many tumors and does not give the guarantee of non-recurrences the tumor after the operation. Different types of B and T cells in the tumor microenvironment attributed to the degree of antitumor immune responses [11,12].

Detailed knowledge of the immune system function has been growing dramatically in the past few years. This has led to the fact that the immune system able to identify different epitopes during cancer development [13]. Antibodies are highly specific agents, and considered an essential part in cancer immunotherapy but, there are still many challenges to reach the full understanding of the useful immunotherapy effect [13]. Anti-tumor capacity of antibodies by modulating tumor antigen-specific immune responses has not well defined yet. Thus, the ever understanding the fundamental mechanisms of the dynamic interactions between the tumor and the immune system have changed the scientific view of tumor [14]. One of the main goals of tumor immunotherapy was the generation of vaccines to stimulate defensive immunity against tumors. This alternative and safe approach of immunotherapy with the use of internal image antigens as vaccine has generated a great deal of interest in recent years [13]. Active immunotherapy is an interesting field as vaccines mostly have an appropriate side effect profile and good tolerance and can be used with other treatments. As well as, these new immune therapies improvement offers many challenges in tumor eradication [15].

Our experiment here was aimed to determine whether Ab2 represent useful antigen to realize active immunotherapy in sheep with cSCC. For this aim, blood samples and tissue specimens were taken for histological and immunological studies. Estimation of tumor cell protein and its antibodies in serum of sheep were done.

## Materials and Methods

### Ethical approval

The experiments were conducted in accordance with the guidelines laid down by the International Animal Ethics Committee and in accordance with local laws and regulations.

### Animals

A total of 19 Ossimi sheep had a mean age of 1-2 years old and 50-70 kg body weight was used for the this study. These sheep were from different localities within Giza Governorate in Egypt, during the period from October 2015 to April 2016. The case history has been taken, and a special examination for the overgrowth location, size, and nature of lesion has been performed. Out of 19 sheep; 14 suffered from cSCC as indicated histopathology in different areas of skin (tail, flank, abdomen, and udder) and five with apparently healthy skin. The five sheep with apparently healthy skin were considered as a control group (G1, n=5). The 14 sheep with cSCC were divided into untreated sheep with cSCC as the positive control group (G2, n=4), sheep with cSCC and treated with Ab1 (G3, n=5) and sheep with cSCC and treated with Ab2 (G4, n=5).

### Preparation of tumor cells and antibodies

Preparation of tumor cell suspension was outlined by Jun *et al*. [16]. Briefly, specimens of tumor tissue were instantly minced in physiological saline and washed several times. The cells were then separated using 60-mesh stainless steel sieves and aspirated by 5-ml pipette. The disseminated, single cell suspension was counted using hemocytometers. The viability of cells was assessed by Trypan Blue exclusion method [17]. The suspension was subjected to sequential low frequency (9 kc/s) sonication in a Raytheon model DF-101 Sonic Oscillator. Half pellets were resuspended and stored for protein measurements while another half was used for the preparation of antibody (Ab1) in rabbit as previously described by Philip and Noboru [18]. Half samples from sera containing rabbit Ab1 were pooled precipitated with ammonium sulfate and extensively dialyzed against 10 mM Tris-HCl buffer, pH 7.5 to isolate the immunoglobulin fraction. Second series of immunization of the fraction was done in the same strain of rabbit as the first regimen to obtain secondary antibody (Ab2).

### Immunotherapy

Each animal from control groups G1 and G2 was treated with a course of eight subcutaneous injections of normal globulins of rabbit sera at several sites of the neck and abdomen. Animals in G3 received a course of eight injections of previously prepared globulin of rabbit Ab1. Each animal in the last group (G4) received a course of eight injections of previously prepared globulin of rabbit Ab2. The animals were injected with five injections with 2 weeks’ interval followed by three injections at 1 week interval [19]. Briefly, the first five injections composed of 0.5 ml of globulin + 0.5 ml complete Freund’s adjuvant (Diffco, Holland) while each of the past three injections composed of 1.0 ml of globulin + 1.0 ml incomplete Freund’s adjuvant.

### Assessing the volume of cutaneous tumors

Each tumor in G3 and G4 was measured before each injection using caliper [20]. The length (L=Longest dimension), the width (W=The distance perpendicular to and in the same plane as the length), and the height (H=The distance between the exterior tumor edge and the sheep body) were measured. To provide the most accurate measure of tumor mass the ellipsoid volume of each tumor was calculated from 0.5 × L × W × H [20]. For multi-lobed tumors or those with irregular shapes, the tumor lobes were measured separately. The volume for each lobe was calculated and summed to obtain the volume of the whole mass.

### Samples collection

Blood samples were collected before the first five injections and after 3 weeks from the past injection. Each sample was divided into two parts. The first half was collected into an ethylenediaminetetraacetic acid containing vacutainer tubes to be used in the determination of total (total leukocyte count [TLC]) and differential leukocyte count. The second half of blood sample was collected in plain tubes and allowed to clot for serum extraction to be used in estimation of tumor cell protein and its antibody. Tissue specimens were collected from all groups after 1 week from the last injection and fixed in 10% buffered formalin for histopathological [21].

### Immunological technique

The potency of Ab1 was determined using indirect enzyme-linked immunosorbent assay (ELISA) [22]. ELISA micro-titer plates (Nunc Co., Denmark) were coated with 100 µl/well of serial concentrations of tumor cell protein dissolved in carbonate bicarbonate coating buffer pH 9.6 and incubated overnight at 4°C. After that, the plates were washed 3 times with washing buffer (phosphate-buffered saline [PBS] pH 7.2 containing 0.05% Tween-20) then blocked with 200 µl/well blocking buffer (gelatin 0.5%, pH 7.2) and incubated for 2 h at 37°C with shaking. Subsequently, plates were washed, and serial double-fold concentrations of Ab1 were added in the plates (dilution/column) then incubated at 37°C for 2 h with shaking followed by 3 times washing. Enzyme conjugate horseradish peroxidase labeled goat anti-rabbit immunoglobulin G (Sigma, USA) 100 µl/well at a titer of 1:4000 dilution in PBS, pH 7.2 was then added and incubated for 1 h at 37°C with shaking. After washing, 100 µl/well of enzyme substrate solution containing ortho-phenyl diamine (Sigma Chemical Co. USA) was added and the plates were incubated in the dark for 7 min at room temperature. The reaction was stopped with 50 µl/well of stopping solution (H_2_SO_4_ 2.5M). Finally, the color reaction was measured at wave length 450 nm using microplate ELISA reader (Bio-Tek, Inc., ELx, 800UV). Estimation of tumor cell protein and its antibodies in serum of sheep was done using the tested samples. Briefly, for estimation of tumor cell protein in serum of sheep, similar steps were performed by coating the ELISA microplate wells with 100 µl tested serum samples. For estimation of Ab1 in serum of sheep, similar steps were performed by coating the ELISA microplate wells with 100 µl tumor cell protein followed by serum samples. For estimation of antibodies against Ab2, similar steps were performed by coating the ELISA microplate wells with 100 µl Ab1 followed by sheep serum samples.

### Leukogram

TLC was done according to Coles [23]. Differential leukocyte count such as monocytes, lymphocytes, neutrophils, and eosinophils were estimated by cross-sectional method [24].

### Statistical analysis

All data were subjected to one-way analysis of variance using GLM procedure. All values were presented as the mean ±pooled standard error. Significant differences were determined by Duncan’s new multiple range.

## Results

### Macroscopic appearance

**Figure-1:**
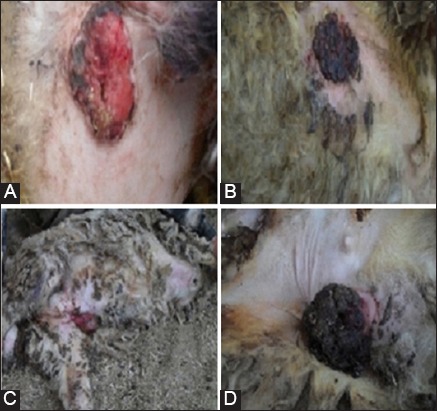
Cutaneous squamous cell carcinoma on the skin of tail (A), flank (B), udder (C), and abdomen (D) of sheep.

Figure-1 showed a variety of different cSCC at one or more sites including the skin of tail, flank, udder and abdomen that was commonly appeared red, scaling, thickened patch on the sun-exposed skin. Macroscopically, the tumor was often elevated, fungating, or might be ulcerated with irregular borders. The symptoms included sores, ulcers, discoloring, and hyperkeratosis in the skin.

### Volume of cSCC

Tumor volume and its regression % in the five Ab2 treated cases were illustrated in Table-1. The decline in the tumor volume started in the 3^rd^ week after Ab2 injection reaching its maximum regression % at the end of the experiment in most cases. It was observed that the tumor volume declined after Ab2 injection and the regression % was 28.13%, 23.59%, 78.12%, 42.96%, and 56.57% in 1^st^, 2^nd^, 3^rd^, 4^th^, and 5^th^ cases at the end of the experiment, respectively. Table-2 summarized tumor volume and regression % in the five Ab1 treated cases. It was observed that the tumor volume declined after Ab2 injection and the regression % were 4.91%, 2.51%, 1.37%, 1.99%, and 3.28% in 1^st^, 2^nd^, 3^rd^, 4^th^, and 5^th^ cases at the end of the experiment, respectively.

**Table-1:** Tumor volume and regression % in five Ab2 treated cases.

Sampling time	Groups									

	Case I		Case II		Case III		Case VI		Case V	
				
	Tumor volume (cm^3^)	Regression %	Tumor volume (cm^3^)	Regression %	Tumor volume (cm^3^)	Regression %	Tumor volume (cm^3^)	Regression %	Tumor volume (cm^3^)	Regression %
1^st^ week	109.80	-	14.67	-	16.73	-	25.56	-	36.13	-
3^rd^ week	110.00	+0.18	12.65	13.77	8.47	49.37	20.55	19.60	26.15	27.62
5^th^ week	99.92	8.99	11.40	22.29	11.77	29.65	18.83	26.44	31.12	13.87
7^th^ week	89.96	18.06	14.67	0.00	12.01	28.21	17.97	29.69	19.49	46.06
9^th^ week	90.43	17.64	12.65	13.77	13.24	20.86	17.84	30.20	19.92	44.87
10^th^ week	97.19	11.48	11.40	22.29	11.83	29.28	17.57	31.26	17.09	52.69
11^th^ week	81.60	25.68	11.48	21.74	9.43	43.63	14.77	42.21	16.57	54.14
12^th^ week	78.91	28.13	11.21	23.59	3.66	78.12	14.58	42.96	15.69	56.57

**Table-2:** Tumor volume and regression % in five Ab1 treated cases.

Sampling time	Groups									

	Case I		Case II		Case III		Case VI		Case V	
				
	Tumor volume (cm^3^)	Regression %	Tumor volume (cm^3^)	Regression %	Tumor volume (cm^3^)	Regression %	Tumor volume (cm^3^)	Regression %	Tumor volume (cm^3^)	Regression %
1^st^ week	23.01	-	98.72	-	112.41	-	19.54	-	27.11	-
3^rd^ week	23.81	+3.48	98.33	0.40	112.59	+0.16	19.38	0.82	27.55	+1.62
5^th^ week	22.66	1.52	97.55	1.18	112.35	0.053	18.57	4.96	27.42	+1.14
7^th^ week	22.87	4.09	97.12	1.62	112.24	0.15	18.92	3.17	25.91	4.43
9^th^ week	20.53	10.78	96.02	2.74	111.24	1.04	19.24	1.54	25.83	4.72
10^th^ week	21.14	8.13	96.19	2.56	111.16	1.11	18.36	6.04	25.40	6.31
11^th^ week	21.96	4.56	94.10	3.40	110.74	1.49	18.19	6.91	26.17	3.47
12^th^ week	21.88	4.91	94.98	2.51	110.87	1.37	19.15	1.99	26.22	3.28

### Microscopical examination

**Figure-2:**
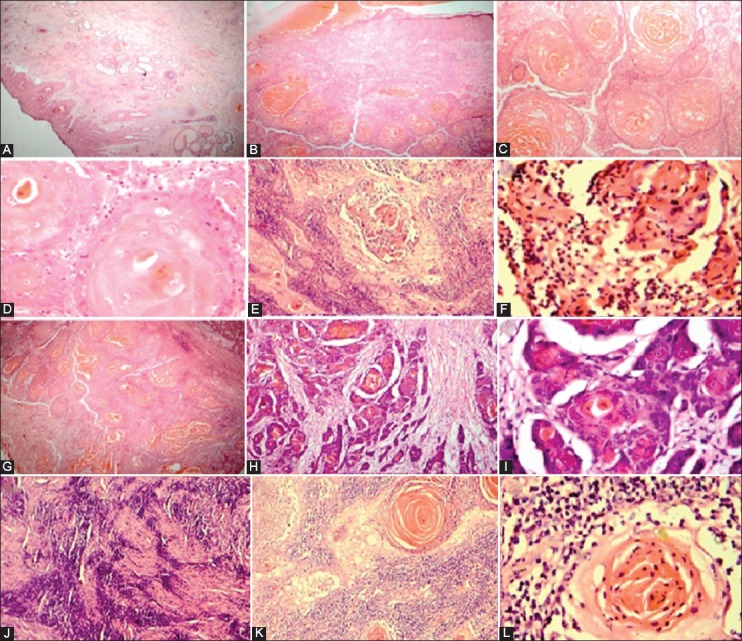
Normal skin of sheep showing dermis and epidermis with various adnexa (H and E, 40×) (A), cutaneous squamous cell carcinoma (cSCC) in the skin of sheep tail showing masses of neoplastic cells forming numerous cell nests of various size. The larger cell nest contained keratinized centers (epithelial pearls) (H and E, 40×) (B), (H and E, 100×) (C) and (H and E, 400x) (D). Excaudate and polymorph infiltration of nests in cSCC in the skin of sheep without immunotherapy (H and E, 40×) (E) and (H and E, 400×) (F). cSCC in the skin of tail of sheep treated with Ab1 showed neoplastic invasion of tumor cells into subcutis forming cell nests with invasion of transformed keratinocytes without polymorph infiltration (H and E, 40×) (G), (H and E, 400×) (H) and (H and E, 40×) (I). cSCC in the skin of tail of sheep treated with Ab2 showed intensive lymphocytic infiltration of nests (H and E, ×40) (J and K) and (H and E, ×400) (L).

Figure-2A showed normal skin of sheep showing dermis and epidermis with various adnexa. Figures-2B:F showed cSCC in the skin of tail and abdomen of cSCC control group. Histological characteristic of cSCC was ranged from well differentiated to poorly differentiated invasive squamous epithelium associated with sclerosis and inflammation. cSCC was characterized by transformation of epidermal keratinocytes resulting in cell nest keratinization through tumor cells. The tumor cells mostly similar those of normal stratum spinosum, but have vesicular nuclei with one or multiple very prominent nucleoli. Higher magnification of cSCC in the skin of abdomen showing some signs of anaplasia such enlargement and hyperchromasia of nuclei, multiple nuclei, and mitotic figures (Figure-2D). Superficial part of cSCC in the skin showing ulceration and inflammatory cellular exudation (Figure-2B:D). Polymorph infiltration was also observed (Figure-2E and F). cSCC in the skin of tail of sheep treated with Ab1 showed neoplastic invasion of tumor cells into subcutis forming cell nests with penetration of keratinocytes through the basement membrane into the dermis without polymorph infiltration (Figure-2G-I). cSCC in the skin of tail of sheep treated with Ab2 showed intensive lymphocytic infiltration of nests (Figure-2J-L).

### Immunological results

Table-3 summarized that tumor cell protein concentration in the serum of cSCC affected sheep was significantly higher than intact skin group throughout the experimental period. In Ab1 treated group, tumor cell protein concentration significantly decreased in the 1^st^ and 2^nd^ weeks then increased in the 7^th^, 9^th^, and 12^th^ weeks comparing to control cSCC affected group. However, tumor cell protein concentration in Ab2 treated group was significantly decreased in the 1^st^ week followed by a significant increase in 5^th^, 7^th^, and 9^th^ weeks comparing with control intact skin group.

**Table-3:** Tumor cell protein in serum of sheep (ng/100 µl).

Sampling time	Groups				

	Healthy	cSCC	Ab1 treatment	Ab2 treatment	Pooled SE
1^st^ week	0.254^d^	2.676^a^	2.031^b^	1.219^c^	0.077
3^rd^ week	0.362^c^	2.309^ab^	1.286^cb^	1.937^ab^	0.425
5^th^ week	0.651^c^	1.515^b^	1.315^b^	2.239^a^	0.084
7^th^ week	0.310^c^	1.769^b^	2.428^b^	2.382^b^	0.201
9^th^ week	0.571^c^	1.665^b^	2.434^a^	2.448^a^	0.145
12^th^ week	0.348^b^	1.879^a^	2.524^a^	2.511^a^	0.214

^abc^Means within a row with no common superscript differ significantly at p<0.05. SE=Standard error, cSCC=Cutaneous squamous cell carcinoma

Table-4 summarized that tumor cell protein antibody in serum of control cSCC affected sheep was significantly higher than intact skin group from the 7^th^ week until the end of the experiment. However, there was a significant increase in tumor cell protein antibody concentrations in Ab1 and Ab2 treated groups than the intact control group in the 9^th^ and 12^th^ weeks of sampling.

**Table-4:** Tumor cell protein antibody in serum of sheep (ng/100 µl).

Sampling time	Groups				

	Healthy	cSCC	Ab1 treatment	Ab2 treatment	Pooled SE
1^st^ week	0.896^b^	0.939^b^	0.345^b^	0.713^b^	0.405
3^rd^ week	0.885	1.175	1.059	0.898	0.371
5^th^ week	0.346^b^	0.346^ab^	0.753^b^	0.881^b^	0.938
7^th^ week	0.565^b^	2.093^a^	1.367^ab^	1.686^ab^	0.603
9^th^ week	0.494^b^	1.472^a^	1.223^a^	1.921^a^	0.207
12^th^ week	0.541^b^	1.429^a^	1.081^ab^	1.663^a^	0.232

^abc^Means within a row with no common superscript differ significantly at p<0.05. SE=Standard error, cSCC=Cutaneous squamous cell carcinoma

Data in Table-5 summarize the estimation of antibody against Ab2 in sera of sheep. The results showed a significant increase in control cSCC affected group starting from the 5^th^ week till the end of the experiment comparing to control with intact skin group. A significant increase in antibody was observed in the Ab2 treated group than in control cSCC affected the group in the 7^th^ and 12^th^ weeks of the experiment.

**Table-5:** Antibody against Ab2 in serum of sheep (ng/100 µl).

Sampling time	Groups			

	Healthy	cSCC	Ab2 treatment	Pooled SE
1^st^ week	0.584	0.874	0.891	0.338
3^rd^ week	0.992	1.167	1.781	0.823
5^th^ week	0.579^b^	1.723^a^	2.440^a^	0.213
7^th^ week	0.331^b^	1.346^a^	2.040^c^	0.242
9^th^ week	0.456^b^	2.167^a^	2.517^a^	0.218
12^th^ week	0.126^c^	1.913^b^	2.373^a^	0.045

^abc^Means within a row with no common superscript differ significantly at p<0.05. SE=Standard error, cSCC=Cutaneous squamous cell carcinoma

### Leukogram results

Table-6 summarized the TLC in healthy and tumor affected sheep blood during immunological treatments. Results showed that total leukocytes increased in cSCC untreated control group than healthy sheep while it was decreased in Ab1 treated sheep in the 1^st^, 3^rd^, and 5^th^ weeks. However, an elevation in TLC was observed in Ab2 treated group than all other groups in the 7^th^ and 12^th^ weeks.

**Table-6:** Total and differential leukocytes of sheep.

Sampling time	Groups					

	Parameters	Healthy	cSCC	Ab1 treatment	Ab2 treatment	Pooled SE
1^st^ week	TLC (10^6^/mm^3^)	9.305^b^	12.103^a^	10.079^b^	10.790^ab^	0.980
3^rd^ week		9.202^b^	12.171^a^	9.253^b^	10.902^a^	0.880
5^th^ week		8.658^c^	11.473^a^	10.325^b^	10.201^b^	0.356
7^th^ week		9.852^b^	9.731^b^	10.352^b^	12.103^a^	0.645
9^th^ week		10.336^ab^	9.764^b^	12.051^a^	11.269^ab^	1.307
12^th^ week		9.031^c^	10.690^b^	9.286b^c^	13.114^a^	1.072
1^st^ week	Lymphocytes %	55.75^a^	50.75^ab^	55.75^a^	55.50^a^	11.066
3^rd^ week		54.25^a^	44.75^b^	57.00^a^	55.75^a^	12.083
5^th^ week		47.00^b^	53.00^ab^	58.00^a^	57.00^a^	2.045
7^th^ week		52.00^b^	51.75^b^	57.75^a^	57.00^ab^	1.216
9^th^ week		52.00^bc^	45.50^c^	61.25^a^	60.25^abc^	3.208
12^th^ week		50.75^a^	48.75^a^	57.25^a^	60.25^b^	4.185
1^st^ week	Monocyte %	3.25	3.50	3.25	3.50	0.433
3^rd^ week		3.25	3.25	3.00	3.50	0.483
5^th^ week		3.50	3.50	2.75	3.50	0.650
7^th^ week		3.50	2.75	3.75	3.50	0.416
9^th^ week		4.25	3.50	4.50	3.75	3.366
12^th^ week		3.75	3.50	4.25	4.00	1.016
1^st^ week	Acidophil %	2.75	3.25	3.50	3.00	0.366
3^rd^ week		3.75	3.25	3.00	3.00	0.433
5^th^ week		3.00	3.50	3.25	3.75	0.483
7^th^ week		3.75^a^	3.50^ab^	2.75^b^	3.25^ab^	0.350
9^th^ week		2.50^ab^	3.25^a^	2.00^b^	2.25^ab^	0.566
12^th^ week		2.75	3.25	2.50	2.25	1.200
1^st^ week	Neutrophil %	41.25^a^	35.50^b^	35.25^b^	34.75^b^	4.00
3^rd^ week		37.75	37.25	34.25	35.75	11.600
5^th^ week		38.75	35.50	33.75	36.25	17.600
7^th^ week		39.75	37.50	36.25	38.00	2.488
9^th^ week		33.00	39.75	32.25	38.00	4.883
12^th^ week		36.75	43.25	41.25	36.75	3.633
1^st^ week	N/L ratio	78.36^ab^	70.01^bc^	63.34^c^	62.80^c^	4.065
3^rd^ week		69.84^bc^	83.28^a^	60.14^c^	64.05^c^	8.427
5^th^ week		83.58^a^	67.12^b^	58.53^b^	63.73^b^	11.614
7^th^ week		76.51^ab^	72.68^ab^	62.73^b^	66.59^ab^	12.574
9^th^ week		63.65^bc^	76.36^a^	62.62^c^	72.09^ab^	13.174
12^th^ week		74.48	89.13	72.38	70.43	17.053

^abc^Means within a row with no common superscript differ significantly at p<0.05. SE=Standard error, cSCC=Cutaneous squamous cell carcinoma

Concerning differential leukocyte count Table-6 summarized that there were a lower lymphocytes values in cSCC control group than healthy in the 3^rd^ week of injection. However, Ab1 and Ab2 treated groups showed elevation of lymphocytes than control healthy and cSCC control sheep. There was no any significant variation in monocyte % between different groups.

Concerning neutrophil% as shown in Table-6, there was a significant decline in cSCC control group and both Ab1 and Ab2 treated groups compared to the healthy control group after the 1^st^ week of the treatment. An elevation in neutrophil lymphocyte ratio (N/L ratio) was recorded in cSCC control group after 3^rd^ and 9^th^ weeks of injection comparing with intact skin control group. Both Ab1 and Ab2 treated groups showed a decline in the ratio after the 1^st^ and 5^th^ weeks of injection comparing with intact skin control group. As well as, Ab1 treated group showed a decline in N/L ratio after the 3^rd^ and 7^th^ injection while Ab2 treated group showed this decline after the 3^rd^ week of injection comparing with cSCC control group.

## Discussion

Skin tumors in sheep, particularly cSCC, have been reported in the literature and identified by some authors as a relatively common lesion in the tropical zones [2,4,25]. In this study, numerous cSCC has occurred in sheep at one or multiple sites including the skin of abdomen, tail, udder, and the flank as previously described by Ahmed and Hassanein [2] and Ahmed *et al*. [26]. Macroscopically, the lesions appeared productive or erosive as recorded previously by Tmumen *et al*. [4] and Webb *et al*. [9]. Ulceration was present probably in many instances as a result of removal of the cutaneous horn by trauma [4]. The emphasized reason of skin tumor is still generally indistinct. However, several predisposing factors are supposed to have a key role in initiating such tumors. One of these factors is the constant exposure to sunlight and ultraviolet radiation [1,2,4]. Based on the geographical location of the sheep enrolled in this study, the constant exposure to radiation is likely to be the predisposing factor. These results were also parallel to those concluded previously by Tmumen *et al*. [4]. Histological characteristic in the present investigation was ranged from poorly to well differentiated invasive epithelium associated with polymorph infiltration as previously recorded by Webb *et al*. [9]. cSCC showed some signs of anaplasia such as enlargement and hyperchromasia of nuclei, and multiple nuclei. Several degrees of keratinization to form cell nests and mitotic figures were observed in sections from the different sites of the skin lesion. These results were in the same line to those results detected previously by Tmumen *et al*. [4], Ahmed *et al*. [26], Baniadam *et al*. [27]. In this investigation, tumor volume and tumor size regression % were measured in the cases of cSCC after Ab1 or Ab2 treatment. In the five Ab2 treated cases, it was observed that the decline in tumor volume as well as the regression % was parallel to each other in most cases and started at the 3^rd^ week of treatment. The percent of regression in Ab2 treated cases was ranged between minimum of 8.99% and maximum of 78.12%, however, the measurements in most cases reached the maximum regression after the past two injections. On the other hand, an increase was observed in the tumor volume of the most Ab1 treated cases at the 3^rd^ week of treatment. The recorded regression % of Ab1 treated cases was ranged between minimum of 0.053% and maximum of 10.78% which was considered low when compared with that of Ab2 treated cases. As well as, the maximum regression % in most Ab1 treated cases was recorded at the 11^th^ week of treatment followed by a decline in regression % at the end of the experiment. These results confirmed that active immunotherapy was aimed to stimulate the immune cells to direct its works toward tumor regression by enforcing immune responses specific for membranous tumor associated antigen presented to phagocytic cells [28,29]. Neutrophils are the first phagocytic cells that have a key role in innate immunity and play a critical role in different inflammatory conditions as a first line of defense [30]. It was observed from our results that there was a significant decline in neutrophil % in cSCC control group and both Ab1 and Ab2 treated groups comparing to healthy control group in the 1^st^ week of the treatment. Thus, it is expected that neutrophils can be mobilized to tumor sites; particularly, it was well known that inflammation has been considered as a key feature of both primary tumor growth and metastasis [31]. Furthermore, accumulation of neutrophils has been reported previously in a broad range of tumors [32]. An interaction between neutrophils and tumor/stromal cells was reported by direct inhibition of tumor proliferation and apoptosis induction through antitumor functions of neutrophils [33]. Neutrophils have a necessary role for survival, progress, and activation of other immune cells to induce immune responses against pathogens [34,35]. It was still doubtful if tumor-associated neutrophils have the capability to enhance antitumor immunity without therapeutic interfering [36,37].

TLC significantly elevated in cSCC control sheep than healthy one during the experimental periods except 7^th^ and 9^th^ weeks. However, in Ab1 treated sheep there was a significant decrease in TLC in the 1^st^, 3^rd^, and 5^th^ weeks of Ab2 injection than cSCC control group. On the other hand, an elevation in TLC was observed in Ab2 treated group in the 7^th^ and 12^th^ weeks comparing to cSCC control sheep. There was a lower lymphocyte % in cSCC control group than healthy in the 3^rd^ week of injection. However, Ab1 or Ab2 treated groups showed elevation of lymphocytes than cSCC control sheep. An elevation in N/L ratio was recorded in control cSCC in the 3^rd^ and 9^th^ weeks of injection comparing with intact skin control group. Both Ab1 and Ab2 treated groups showed decline in the ratio after the 1^st^ and 5^th^ weeks of injection comparing with intact skin control group. As well as, Ab1 treated group showed decline in ratio after the 3^rd^ and 7^th^ injection while active immunotherapeutic treated group showed this decline after the 3^rd^ week of injection comparing with cSCC control group. The overall pictures indicated elevation of lymphocyte as results of injection of Ab2. Histological examination also revealed intensive lymphocytic infiltration at the sites of cSCC lesions after the treatment with Ab2. This infiltration might be a part of immune system reaction with the tumor cells [38]. High densities of tumor-infiltrating lymphocytes have been associated with good prognosis in many tumor types [39]. There was increasing evidence that lymphocyte infiltration as a result of Ab2 treatment was correlated with improved response to chemotherapy in solid tumors as recorded previously by Lee *et al*. [40], Jacobsen *et al*. [41] suggested some sort on B-cell regulation by Ab2 as well as some interleukins might be the principle for lymphocytic infiltration at the site of tumor lesion [42]. Therefore, histological results added confirmation that Ab2 could act as a surrogate antigen.

It was observed from our results that there was a time relationship between immunotherapy by Ab2 and regression of tumor lesions as outlined previously [43-45]. This regression might be attributed to the enhancement of apoptosis than proliferation by immunotherapeutic treatment as previously described by Beresford *et al*. [46], Railo *et al*. [47]. In this study, tumor cell protein concentration in the serum of cSCC control group was significantly higher than healthy group throughout the experimental period. However, it was lower in both Ab1 and Ab2 treated groups comparing to cSCC control group in the 1^st^ week that might be an indication of protein neutralization in the blood of treated animals. Antibodies were highly specific tools, and knowledge of their immune characteristics played an increasingly important role in cancer treatment [13]. It was reported that Ab2 itself could induce antibodies that bind the original tumor-associated antigen, therefore, Ab2 could be used as a surrogate antigen instead of tumor cells [48]. In this study, Ab1 in the serum of control cSCC affected sheep was significantly higher than healthy group from the 7^th^ week until the end of the treatment period. However, Ab1 increased in both Ab1 and Ab2 treated groups in the 9^th^ and 12^th^ weeks. Estimation of and/or treatment with the Ab1 was of great importance as an indication of the elimination of disease. The use of Abs in tumor treatment increased in the recent years [49]. Abs could mediate signaling by tumor associated surface antigen that might lead to destruction of tumor cells and might also alter the cytokine levels or enhanced an antitumor immune response [50,51]. Likewise, Abs had indirect effects included recruiting cells that exert antitumor antibody (Ab)-dependent cytotoxicity [52]. In addition, Abs could also bind complement, leading to complement-dependent cytotoxicity [53] and modified the tumor microenvironment by inhibiting angiogenesis [54]. It was also found that different types of B and T cells in the tumor microenvironment attributed to the degree of antitumor immune responses against self-antigen [11,12].

A significant increase in antibody against Ab2 was observed in control cSCC group starting from the 5^th^ until the end of the experiment comparing to healthy group. In addition, a significant increase in antibody against Ab2 was recorded in Ab2 treated sheep comparing to control cSCC affected sheep in the 7^th^ and 12^th^ weeks of the experiment. In accordance with the idiotypic network hypothesis, the infused Ab1 might elicit an Ab2. Part of the Ab2 response with internal image characteristics might subsequently induce a humoral and cellular anti-idiotype response, Ab3 [55]. It was noticed previously that there was a high specificity of the Ab2 immunization approach after the demonstration of the sequence homology between Ab1 and Ab3, where Ab1 was stimulated by tumor antigen, and Ab3 was stimulated by Ab2 [56]. Ab2 that bind to Ab1 might functionally mimic the tumor-associated antigen defined by the Ab1 [48,57]. It was indicated that the Ab2-induced anti-tumor immunity played a major role in the destruction of metastatic tumor cells [43]. The overall results of immune measurements indicated that Ab2 could be used as a surrogate antigen.

Old therapy for cSCC by surgery or by the use of tumor cell as an antigen has been described previously. Now, a new therapy is the use of Ab2s as antigen. The antigenic mimicry of internal image Ab2 makes them valuable not only as immunogens for pathogens but also for immune therapy for tumors. However, the development of this new immune therapy offers many challenges and need investigation.

## Authors’ Contributions

FAMA designed the study and contributed to experiments performance, laboratory work analysis and data interpretation, manuscript preparation and corresponded the authorship. AAZ contributed to experiments performance, revised the manuscript and further assisted in the discussion. SSAE shared in experiments performance and manuscript review. ASA and AE shared in some experiments performance. All authors have read and approved the final manuscript.
